# THE RELATIONSHIP BETWEEN TECHNOLOGY USE, EMPLOYMENT STATUS, AND SOCIAL ENGAGEMENT IN OLDER AMERICANS

**DOI:** 10.1093/geroni/igae098.2568

**Published:** 2024-12-31

**Authors:** Jaesung Lee, Jay O’Shields, Allison Dunnigan

**Affiliations:** University of Georgia, Athens, Georgia, United States; University of Georgia, Athens, Georgia, United States; University of Georgia, Athens, Georgia, United States

## Abstract

Previous studies have explored the relationship between technology use or employment status among older adults and social engagement. However limited knowledge exists regarding the relationship between technology use, employment status, and social engagement. This study analyzes the relationship between older adults’ information and technology (ICT) use and employment status on social engagement. The 2022 (wave 12) National Health & Aging Trends Study (NHATS) was used. The sample population includes US Medicare beneficiaries aged 65 years and older who were community dwellers (N = 5,195). Multivariate regression analysis indicates that ICT use is associated with social engagement (b=-1.52, t=-11.86, p< 0.001), with ICT users having the highest level of social engagement followed by CT users, IT users, and nonusers. Employment status is also associated with social engagement (b=-0.46, t=-5.14, p< 0.001), with the employed being the most socially engaged, and the retired/do not work anymore group having higher levels of social engagement compared to the unemployed group. An interaction was tested but was not significant. Additionally, sociodemographic factors including age (b=0.05, t=2.50, p=0.015), gender (b=0.31, t=5.71,p< 0.001), race (b=-0.78, t=-9.52, p< 0.001), education(b=0.24, t=3.70, p< 0.001), and living arrangements (b=0.31, t=6.84, p< 0.001) have a statistically significant relationship with social engagement. In conclusion, this study emphasizes the importance of considering both technology use and employment status in understanding social engagement among older Americans. These findings suggest the need for further research to explore the complex interaction of these factors in older adults’ social engagement.

